# Influence of diamond particle size on the properties of diamond/Al composites fabricated by filtration extrusion

**DOI:** 10.1016/j.heliyon.2024.e37391

**Published:** 2024-09-03

**Authors:** Junfeng Zhao, Shuohang Yun, Qiulin Li, liang Wang

**Affiliations:** a Guangdong Provincial Key Laboratory of Electronic Functional Materials and Devices, Huizhou University, Huizhou, 516001, China; b Joint Laboratory of Nuclear Materials and Service Safety, Shenzhen International Graduate School, Tsinghua University, Shenzhen, 518055, China; c Shenzhen Zhixing New Materials Technology Co.,Ltd, Shenzhen, 518055, China

**Keywords:** Diamond/Al, Filtration extrusion behaviors, Organizational evolution

## Abstract

High-content diamond/Al composites have great potential for application as heat dissipation substrates in high-power electronic devices due to their superior thermal properties. This study addresses the persistent challenge of efficiently and economically preparing high-content diamond/Al composites. The filtration extrusion technique as a novel approach for fabricating these composites and explore the critical impact of diamond particle size on the extrusion process and the resulting microstructures. Our findings reveal that extrusion force remains minimal at the onset of the process but escalates sharply with an increase in diamond content, peaking upon completion of extrusion. Notably, larger diamond particles precipitate a more abrupt rise in extrusion force during displacement. The composites' density and thermal conductivity exhibit an initial increase followed by a decline as the diamond particle size is incremented, while the thermal expansion coefficient shows a progressive rise with size enlargement. These insights are pivotal for optimizing the fabrication parameters to achieve high-performance diamond/Al composites for thermal management applications.

## 1 Introduction

In recent years, the problem of thermal failure of electronic components has become an obstacle to the sustainable development of the electronics industry [1,2]. Improving the thermal performance of packaging thermal management materials has become a research hotspot in this field [3,4]. Based on the needs of electronic information technology, the heat dissipation materials have been developed for multiple generations, such as Invar alloy, Kovar alloy, W-Cu alloy, SiC/Al and so on[5,6]. The diamond/metal composite materials are of great interest in the field of thermal management owing to the excellent thermophysical properties [7,8]. Among them, the high-content diamond/Al composites have received more attention due to their relatively lower densities and high thermal conductivity. Therefore, diamond/Al composites considered to be the most promising materials for high-performance electronic devices. The current methods for preparing the diamond/Al composites include hot press sintering [8], discharge plasma sintering [9], force infiltration [10], forceless infiltration [11] and so on [12,13]. Some scholars have also studied other types of metal matrix composites, such as Cu/diamond composites prepared using hot-forging [14,15], which have achieved good thermal performance. However, the efficient and economical preparation of high-volume-fraction diamond/Al composites has always been a research challenge.

The thermal performance of diamond/Al is greatly dependent on a variety of parameters in the process, especially the volume fraction of diamond [[16], [17], [18]]. Some scholars are prepared the diamond/Al composites with different diamond content and focus on explore the effect of diamond content on the performance of composites [19,20]. Referring to the previous research [[21], [22], [23]], it has been observed that incorporating a high-volume-fraction diamond in the composites can aid in achieving high thermal conductivity (TC) and an ideal coefficient of thermal expansion (CTE) that matches with the chip. Monje et al. adopted the force infiltration to fabricate the diamond/Al composites and improved the thermal conductivity of the composites by controlling the infiltration temperature and contact time [24]. But the diamond/Al composites prepared by liquid phase infiltration are prone to have voids that cannot be filled by the Al metal matrix. Increasing the diamond content in diamond/Al composites prepared using powder metallurgy can make it difficult to press form the mixed powder, resulting in a significant reduction in density, which further leads to a sharp decrease in thermal conductivity. Due to the limitations of liquid phase infiltration and powder metallurgy, there is a need for alternative methods to produce diamond/Al composites with high volume fraction of diamond [25,26]. Hence, the development of efficient and cost-effective approaches for preparing high-content diamond/Al composites has become a challenging research area.

The filtration extrusion technique utilizes a thixoforming process of semi-solid metals to effectively separate liquid and solid phases under force, which offers distinct benefits such as near-net-shape production and a brief preparation cycle. This technique is superior to other methods and results in high volume fraction of diamond in diamond/Al composites [27]. Zhou et al. proposed the liquid-solid separation technique, a compact processing routine to fabricate high-performance diamond/Al composites [28]. Guo et al. obtained the SiC/Al composites with high volume fraction of SiC as the electronic packaging shell through liquid-solid separation technique [29]. Filtration-extrusion technology is low-cost and easy to fabricate high-performance diamond/Al composites which can realize near-net-shape fabrication [27]. Hence, while the filtration extrusion technique offers a feasible method for producing high-content diamond/Al composites, further investigation is needed to understand the impact of extrusion behavior on the performance of the final product.

This paper designed a filtration extrusion device and used low-content diamond/Al homogeneous composites as experimental materials to explore the feasibility of preparing high-content diamond/Al composites using filtration-extrusion technique. The effects of diamond particle size on the filtration extrusion behavior and property of the composites were studied.

## 2 Experimental

### 2.1 Materials

As the matrix material, pure Al powers with an average particle size of about 30 μm were purchased from Beijing HuiSheng New Materials Technology Co.,Ltd. in China. As the reinforcement material, diamond particles with four sizes were manufactured by Henan Huanghe Whirl Wind Co., Ltd. in China. The manufacturers and particle sizes of raw materials are presented in Table 1. The chemical composition of commercial Al powders is shown in Table 2.

**Table 1:** Experimental raw materials and manufacturers.

Materials	Particle size (μm)	Manufacturer
Commercial pure Al powder	30	Beijing HuiSheng New Materials Technology Co.,Ltd
Diamond particles	40/80/200/400	Henan Huanghe Whirl Wind Co.Ltd

**Table 2:** Chemical composition of commercial Al powders.

Components	Al	Mg	Fe	Si	Ga	Na	Others
Content (wt.%)	99.72	0.123	0.0696	0.0246	0.0171	0.0122	0.0313

### 2.2 Preparation of the high-content diamond/Al composites

The experimental preparation process was as follow: powder mixing, cold press forming, and filtration extrusion.

**Fig. 1:**
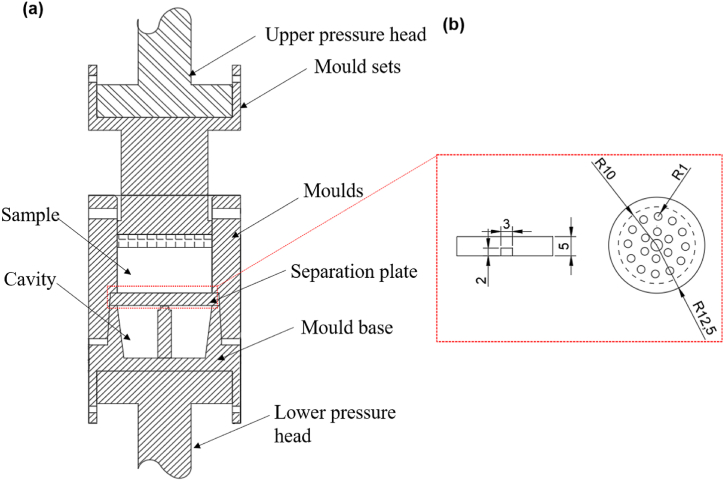
(a) The schematic diagram of the structure of the filtration-extrusion; (b) Schematic diagram of the separation plate structure(mm).

(1) Powder mixing: A blend of 20 vol percent diamond particles and aluminum powders was thoroughly mixed using a non-Intrusive Material Homogenizer. The mixing procedure involved a revolution speed set at 20 rs^−1^ and a rotation speed of 16 rs^−1^, maintaining a 5:4 ratio between the rotation and revolution speeds. To ensure a controlled environment, a vacuum pump was engaged at the onset of the mixing, drawing the mixing chamber's force down to approximately 0.1 KPa. The mixture was agitated for a precise duration of 200 s before the vacuum was deactivated, halting the blending process. The combined powder was then conveyed into a vacuum drying chamber, where it was dehumidified for a full 24 h. Finally, the homogenized blend of diamond and aluminum powders was sealed under vacuum to reduce oxygen contact, ready for the subsequent cold pressing stage.
(2) Cold press forming: The vacuum-sealed powder was removed and loaded into a hard alloy mold with a diameter of 20 mm. A force of 500 MPa was applied using the manual cold press machine, and the force was held for 1 min. After demolding, a cold-pressed sample with a diameter of 20 mm and a height of 12 mm was obtained. The sample was vacuum-sealed to minimize exposure to oxygen.
(3) The filtration extrusion process:The schematic diagrams of the experimental setup designed and constructed for the preparation of high-content diamond/Al composites using filtration extrusion technology are shown in Fig. 1. The equipment used for the experiment is the Landmark high-temperature electro-hydraulic servo testing system, which is equipped with a resistance furnace for control temperature from room temperature to 1400 °C. The heating power of the resistance furnace is regulated by feedback from two thermocouples on the outside of the mold. The sleeve is sealed using a graphite gasket. The sealing process involves heating the mold to 50 °C higher than the maximum temperature in the experiment, and then cooling the graphite gasket with the sleeve. The seal is achieved by utilizing the thermal expansion and contraction of the mold, preventing the liquid metal melt from leaking out through the gaps between the mold sleeve and the upper plunger during the experiment. The separation plate structure is a circular disk with a diameter of Ф25 × 5 mm, with several small through-holes of the same diameter (Ф2 mm) on it. The cold pressed sample was first heated to 300 °C for 2 min, then to 600 °C for 5 min, finally to a liquid-solid state at 720 °C for 20 min. During the filtration extrusion process, the lower plunger drives the entire mold upward, exerting force on the experimental sample. In this process, the molten aluminum matrix metal flows out from the small through-holes in the separation plate. To ensure that the separation plate does not deform or bend at high temperatures, a supporting column is designed below it. The upper and lower plungers are clamped on the hydraulic press, and parameters such as extrusion force and speed are set and controlled by a computer program connected to the hydraulic press. The molten Al matrix extruded during the extrusion process and accommodated in the cavity. The extrusion rate was 0.1 mm/s during the filtration extrusion process.

### 2.3 Characterization

The microstructures of the diamond/Al composites were characterized by a Micro-Nano Tomography Scanner (Micro-CT, Werth TomoScope, Denmark) and SEM (S-4800, HITACHI). The densities (ρ) of the fabricated composites were determined by Archimedes drainage method. The thermal diffusivities (α) of the diamond/Al composites with size of Φ12.7 mm × 3 mm were tested by an LFA447 laser flash thermophysical machine (NETZSCH, Germany) at room temperature. The thermal conductivity (TC) can be obtained from the formula: λ=ρ×α×Cp.The coefficients of thermal expansion (CTE) of the diamond/Al composites with size of 10 mm × 10 mm × 5 mm were measured by a TMA/SDTA 2+ thermomechanical Analyzer (METTLER-TOLEDO) over a temperature range of 25–220 °C at a heating rate of 5 °C/min. The samples prepared by filtration extrusion were successively mechanically polished on the surface with sandpaper of 400#, 600#, 1000#, and 1500# grit. After being ultrasonically cleaned in ethanol and dried, the density of the samples was tested using the Archimedes drainage method. For each parameter, three samples were taken and the average value was used to represent the numerical value for this specimen.

## 3 Results and discussions

### 3.1 Extrusion force variation during filtration extrusion

**Fig. 2:**
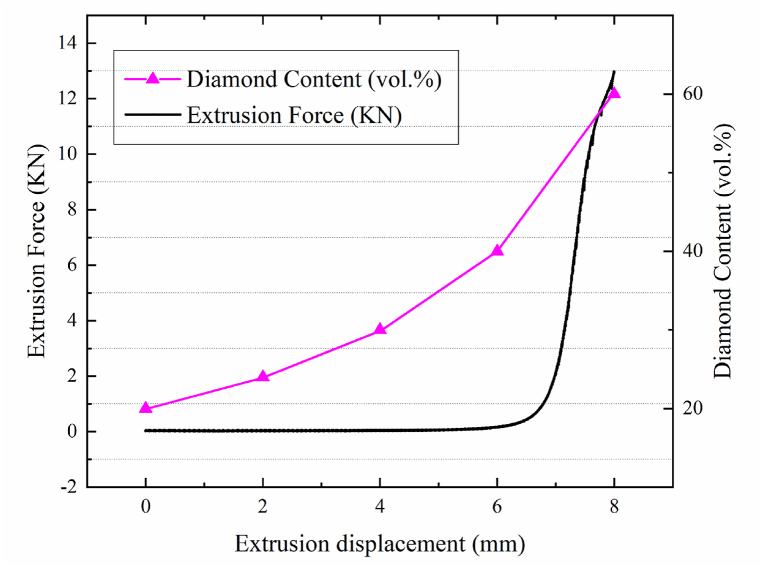
The relationship between force and diamond content (a) 2 mm; (b) 4 mm; (c) 6 mm; (d) 8 mm.

During the filtration extrusion process, changes in the microstructure of composite materials lead to corresponding variations in extrusion force. Therefore, studying the changes in extrusion force aids in a better understanding of the redistribution behavior of composite material microstructures. The variation of the extrusion force with the extrusion displacement and the change in diamond content during the extrusion process are shown in Fig. 2. As the extrusion displacement increases, the extrusion force exhibits a monotonically increasing trend, with the diamond content gradually increasing. Below 6 mm of extrusion displacement, the extrusion force is minimal. However, as the extrusion displacement increases from 6 to 8 mm, the extrusion force rises rapidly, and the volume fraction of diamonds also increases. In the filtration extrusion process, the trend of extrusion force change is closely related to the increase of extrusion displacement. As the extrusion proceeds, the content of diamond particles gradually increases, which elevates the viscosity of the slurry and also increases its rheological force, making the flow of the slurry more difficult. Consequently, a greater extrusion force is required to maintain a constant speed of extrusion. During the extrusion process, diamond particles tend to aggregate near the separation mold. As the extrusion displacement increases, the concentration of particles in these aggregation areas rises rapidly, leading to the formation of a filter cake at the bottom. This cake increases the difficulty for the metal liquid to pass through, necessitating the application of greater extrusion force to ensure the smooth progression of the filtration extrusion. Furthermore, as the liquid phase content in the semi-solid composite material decreases, the distance between diamond particles diminishes, and the increase in frictional forces between the particles surpasses the friction between the particles and the liquid phase. This escalation in frictional forces contributes to a steep rise in the extrusion force trend.

### 3.2 Microstructures evolve with extrusion displacement

**Fig. 3:**
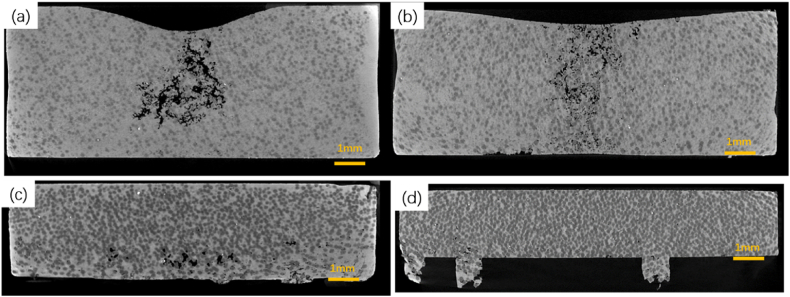
The microstructure of the cross-sectional at different extrusion displacements (a) 2 mm; (b) 4 mm; (c) 6 mm; (d) 8 mm.

Fig. 3 presents the microstructural cross-sections at various extrusion displacements, with (a-d) representing 2 mm, 4 mm, 6 mm, and 8 mm, respectively. Fig. 3(a)–3(c) display a higher incidence of voids within the composites, whereas Fig. 3(d) shows a substantial reduction in void formation. During the initial 0–6 mm of the extrusion process, the applied forces were relatively low (less than 0.5 kN), which failed to facilitate the molten aluminum liquid to fully infiltrate the spaces between the diamond particles. Consequently, as the aluminum matrix contracted during solidification, the upper region of the sample experienced depression, leading to casting defects. The presence of voids could also be attributed to the high volume fraction of aluminum melt (over 50 vol%) in the 0–6 mm range, resulting in increased shrinkage during solidification. Upon reaching an extrusion displacement of 8 mm, the squeezing pressure surged to 13 kN, with the solidification force also increasing in tandem, leading to a significant reduction in casting defects. Moreover, with extended extrusion displacement, there was a notable increase in diamond content and a corresponding decrease in the aluminum matrix content. This compositional shift resulted in diminished volume shrinkage of the sample and a marked decrease in void formation. The augmented extrusion pressure also served to enhance the flow of the molten aluminum and its plastic deformation, allowing it to better penetrate the interparticle spaces and thereby strengthen the interparticle bonding.

**Fig. 4:**
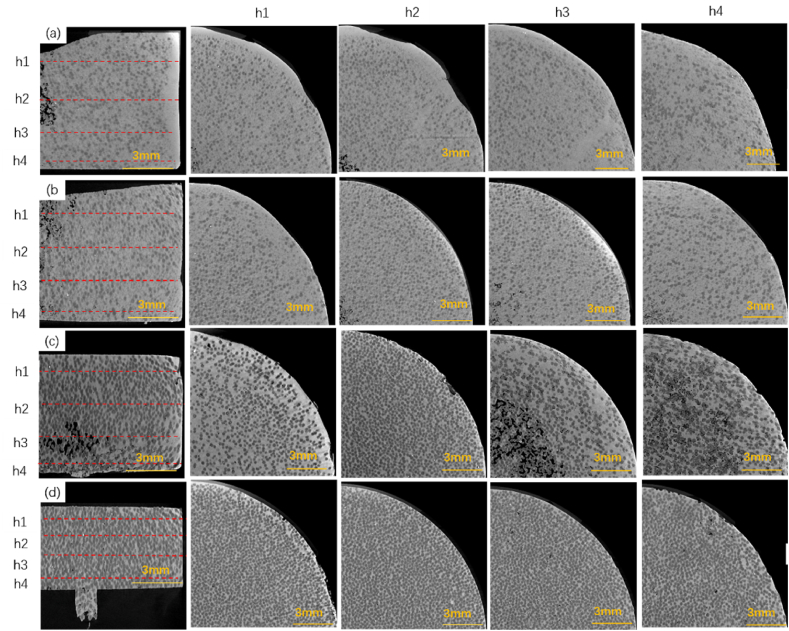
Cross-sectional views for different displacements (a) 2 mm; (b) 4 mm; (c) 6 mm; (d) 8 mm.

Fig. 4 displays cross-sectional images at extrusion displacements of 2 mm, 4 mm, 6 mm, and 8 mm, revealing how the diamond particles densify at different heights as the extrusion progresses. Notably, at a displacement of 8 mm, the diamond particles exhibit a more uniform and dense distribution across all heights compared to the 0–6 mm range. The visual evidence suggests that augmenting the extrusion displacement promotes a more intimate integration between the aluminum matrix and diamond particles. This, in turn, leads to a progressive decrease in casting defects and a concomitant increase in relative density. Such defects are known to significantly impair the thermal conductivity and mechanical integrity of the diamond/Al composites [30]. As depicted in Fig. 4(d), at an 8 mm displacement, the aluminum matrix effectively fills the interstitial spaces between the diamond particles, markedly minimizing the defect count. The findings underscore that extending the extrusion displacement mitigates solidification shrinkage and enhances the diamond volume fraction within the composites.

**Fig. 5:**
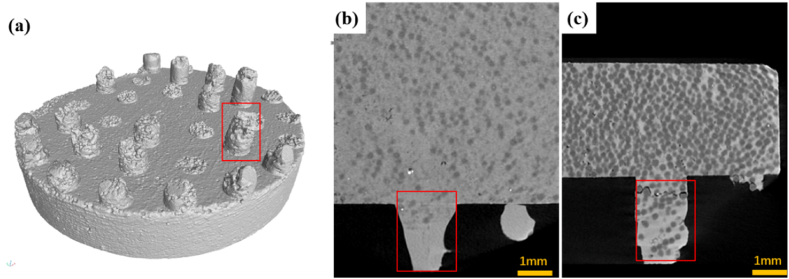
Diamond distribution near separation holes (a) separation plate; Different extrusion displacements:(b) 2 mm; (c)8 mm.

Under the application of extrusion force, diamond particles are partially pressed into the separation holes, as depicted in Fig. 5, which illustrates the distribution of diamonds adjacent to the separation holes at varying extrusion displacements. At a displacement of 2 mm, the separation channels are virtually devoid of diamond particles. The channels' structure is bifurcated: diamond particles are present near the channel's entrance, while the regions further from the entrance are composed solely of the aluminum matrix. With an increase in extrusion displacement, the extrusion force escalates, resulting in an enriched concentration of diamonds within the separation channels. Nonetheless, the channel's interior diamond content remains substantially lower compared to the diamond concentration near the top of the separation channels. The findings indicate a direct correlation between the diamond content within the separation channels and both the applied force and the displacement. At lower extrusion forces, the channel interiors contain fewer diamonds, facilitating the filtration extrusion process. Conversely, as the extrusion force intensifies, an increased number of diamond particles are transported into the separation channels.

### 3.3 The interaction between extrusion force and diamond particle sizes

**Fig. 6:**
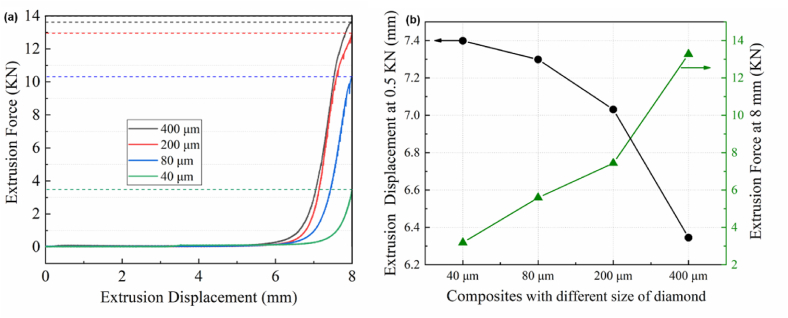
(a) Extrusion displacement versus force; (b) Extrusion displacement at 0.5 KN and force at 80 s.

Fig. 6 shows the extrusion displacement-force curves for samples featuring different diamond particle sizes. During the extrusion process, each sample's extrusion force curve followed a comparable pattern of ascent. For a consistent extrusion displacement of 8 mm, the relationship between extrusion displacement and force for composites crafted with varying diamond particle sizes demonstrated a parallel trend. The initial displacement at which the extrusion force experienced a steep increase diverged with the size of the diamond particles. An upsurge in diamond particle dimensions correlated with an advanced extrusion displacement needed for a significant escalation in extrusion force, concomitantly raising the peak extrusion force at 8 mm. Composite materials with different diamond particle sizes exhibit varying extrusion forces at an extrusion displacement of 8 mm, with larger particle sizes requiring greater extrusion forces. Additionally, the displacement at which extrusion force increases rapidly differs for composites of varying particle sizes; the larger the particle size, the greater the displacement required for the extrusion force to begin its significant increase. When the extrusion force reaches 0.5 KN, the extrusion displacement decreases with the increase in diamond particle size.

The reasons for the changing trend of extrusion force with different diamond particle sizes can be explained from the following three aspects: 1) As the particle size increases, the interparticle interaction forces also grow, leading to an increase in friction between diamond particles. This necessitates a greater extrusion force to push the particles through the filtration channels; 2) With the enlargement of particle size, the difficulty for diamond particles to pass through the filter pores increases. Larger particles require a higher force to ensure the continuous progression of extrusion; 3) Particles with larger diameters typically exhibit poorer fluidity, which in turn increases the extrusion force required for particle movement.

The interaction force between diamond particles during filtration extrusion can be explained by the lubricating force model [31,32]. When the particle spacing ζ reached ζ_min_≤ζ ≤ 2h, the liquid between the particles was squeezed out to generate a lubricating force that hindered the movement of the particles (2h ≈ 8 % Dp, (i)). In the interval of 0 ≤ ζ≤ζ_min_, the lubricating force was not enough to prevent the particle collision, and the dry collision occurred. When ζ_min_≤ζ ≤ 2h, the particle movement was impeded by the lubricating force, resulting in energy loss. The calculation formula is as follow:

(1) $$F_{\text{lub}}=‐\frac{6\pi \mu \rho_{f}\left(u_{f}‐u_{p}\right)}{\zeta }({\frac{D_{p,\left(i\right)}D_{p,\left(j\right)}}{2(D_{p,\left(i\right)}+D_{p,\left(j\right)}})}^{2}n_{p},\zeta_{\min}\leq \zeta \leq 2h$$

where Dp, (i), Dp, (j) are the diameters of particles i, j; ζ is the distance between particles; ζ_min_ is the peak value of particle surface roughness; μ is the apparent viscosity of the fluid; μ_f_ is the fluid velocity and μ_p_ is the particle velocity; n_p_ is the unit basis vector of the collision between particles. When the extrusion displacement was short, the distance between the diamond particles in the Al matrix was large (ζ > 2h), the movement of the particles was not affected by the lubricating force, and extrusion force was small. As the extrusion displacement increased during the filtration process, the diamond particles became more densely packed. When ζmin≤ζ ≤ 2h, the movement of diamond particles was impeded by the lubricating force, requiring a greater force to maintain their motion. During the final stage of extrusion, the spacing between diamond particles became small, resulting in increased resistance to their movement. This led to a rapid increase in extrusion force until the end of the extrusion process.

### 3.4 Microstructure and properties with varying diamond particle sizes

#### 3.4.1 Micrtostructure

**Fig. 7:**
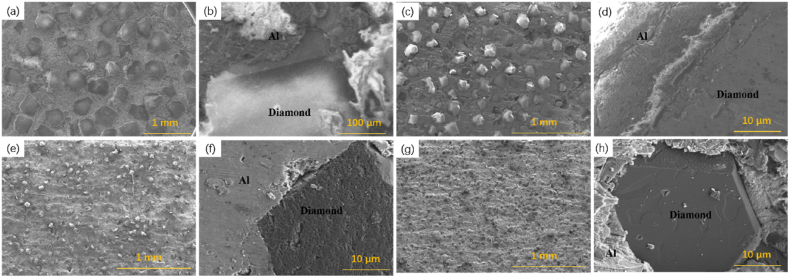
Microstructure of diamond/Al composites with different particle sizes. (a, b): 400 μm-diamond; (c, d): 200 μm-diamond; (e, f): 80 μm-diamond; (g, h): 40 μm-diamond.

The study investigated the impact of diamond particle size on the microstructure and properties of 60 vol%-diamond/Al composites prepared with an extrusion displacement of 8 mm. Fig. 7 shows the microstructure of diamond/Al composites with varying diamond particle sizes. Diamond particles are densely distributed within the Al matrix without evident agglomeration. As the diamond particle size decreases, the quantity of diamond particles in the composite increases, implying a greater dispersion of diamond particles within the same volume of aluminum matrix. With the reduction in diamond particle size, the Al matrix binds more tightly around the diamond particles. The smaller diamond particle size may contribute to the reduction of pore and defect formation, thereby enhancing the density and overall performance of the material. However, no reaction phase was observed at the interface between the diamond and the Al matrix. This is due to the poor wettability of diamond with Al, which prevents the formation of metallurgical bonds between diamond and Al. Consequently, micro-voids are present at the interface of the composite material. These voids lead to an increase in interface thermal resistance, which in turn has a negative impact on the overall performance of the material.

#### 3.4.2 Density

**Fig. 8:**
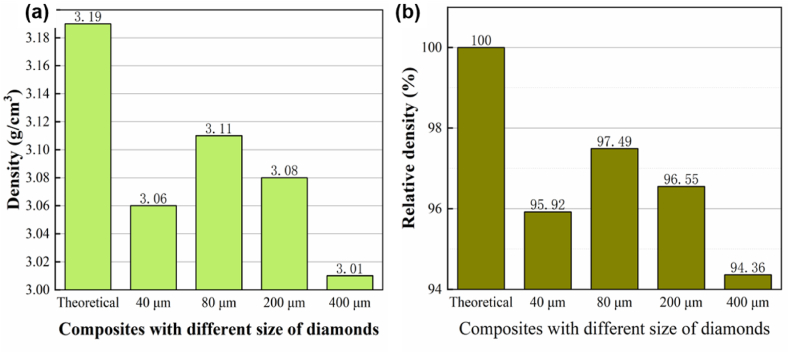
Diamond/Al composites with different particles size of diamond. (a) density; (b)relative density.

Fig. 8 shows the densities and relative densities of diamond/Al composites with different particles size of diamond. The actual density of the composite increased first and then decreases with the increase of diamond particle size. The composite with 80 μm-diamond reached the maximum density of 3.11gcm^−3^, while the density composite with 400 μm-diamond reduced to only 3.01gcm^−3^. The composites with 40 μm-diamond has lower density may be due to the fact that the particles were too small to agglomerate easily and has large specific surface area which mean that more melt is needed with an equal amount of diamond [33]. The relative density of the composite represents the densification of the composite. The relative density of the composites demonstrated similar trend with the densities, which is due to that the difficulty for the rearrangement of particles increased as the particle size increases, leading to the decrease of bulk density. Thus, diamond particles with appropriate sizes are beneficial to sintering densification of the composites.

#### 3.4.3 Thermal conduction

The commonly used theoretical models for thermal conductivity prediction include the Mexwell-Eucken model and the DEM model. The Mexwell-Eucken prediction model is shown in Equation (2), and the DEM prediction model is shown in Equation (3) [34]:

(2) $$\lambda =\frac{\lambda_{Al}\left[2\lambda_{Al}+\lambda_{diamond}+2\left(\lambda_{dia\;\mathrm{mod}}-\lambda_{Al}\right)V_{\text{diamond}}\right]}{2\lambda_{Al}+\lambda_{diamond}-\left(\lambda_{diamond}-\lambda_{Al}\right)V_{diamond}}$$

(3) $$\sqrt[{3}]{\frac{\lambda }{\lambda_{Al}}}\left(1-V_{diamond}\right)=\frac{\frac{\lambda_{diamond}}{\lambda_{Al}}-\frac{\lambda }{\lambda_{Al}}}{\frac{\lambda_{diamond}}{\lambda_{Al}}-1}$$

where λ is the thermal conductivity of the composite, λ_Al_ is the thermal conductivity of the Al matrix, λ_diamond_ is the theoretical thermal conductivity of diamond, and *V*_diamond_ is the volume fraction of diamond.

Due to the particle size of diamond has effects on the interface thermal resistance, the thermal conductivity of composites will be affected [35,36]. The converted effective thermal conductivity λ′_diamond_ is used instead of λ_diamond_.

(4) $$\lambda^{\prime }=\frac{\lambda_{diamond}}{1+\frac{\lambda_{diamond}}{rh_{c}}}$$

where r is the size of diamond particle; h_c_ is the interface thermal conductivity between the Al matrix and the diamond particles, and its expression is:

(5) $$h_{c}=\frac{1}{4}\rho_{Al}c_{Al}C_{Al}\eta_{Interface}$$

where ρ_Al_ is the density of Al, c_Al_ the specific heat capacity of Al, C_Al_ the phonon propagation velocity of Al, and η_Interface_ the phonon heat dissipation coefficient between Al and diamond, which can be determined by the phonon mismatch model as in equation (6):

(6) $$\eta_{Interface}=\frac{2Z_{Al}Z_{diamond}}{{\left(Z_{Al}+Z_{\text{diamond}}\right)}^{2}}\frac{C_{DAl}}{C_{Ddiamond}}$$

Z_Al_ and Z_diamond_ are the phonon impedances of Al matrix and diamond, respectively (Z = ρC_D_, C_D_ is the Debye sound velocity), and C_Ddiamond_ is the phonon velocity of diamond. The phonon velocity of Al matrix and diamond is calculated by Equation (7):

(7) $$C_{D}=\frac{1}{\sqrt{\frac{1}{2}\left(\frac{1}{C_{1}^{2}}+\frac{1}{C_{t}^{2}}\right)}}$$

C_1_ is the phonon velocity passing through the matrix in the longitudinal direction, and C_t_ is the phonon velocity passing through the matrix laterally.

**Fig. 9:**
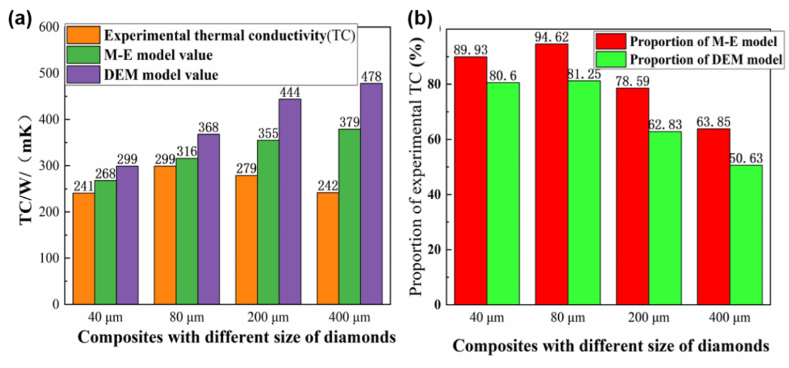
Measured and modelled of thermal conductivity with different particle sizes of diamond.

Fig. 9 demonstrates the comparison between the experimental value and the model predicted value of 60 vol %-diamond/Al composites with different particle sizes of diamond. It can be seen that the thermal conductivities of both the Mexwell-Eucken and DEM models increased as the particle size increased, but the Mexwell-Eucken model values were lower than the DEM values. The thermal conductivity of the experimental sample was basically below 300 Wm^−1^K^−1^. With the increase of diamond particle size, thermal conductivity of the composites increased first and then decreases. Meanwhile, experimental thermal conductivity value was closer to the predicted value of the Mexwell-Eucken model. For the 80 μm-diamond/Al composite, the experimental thermal conductivity reached a maximum value of 299 Wm^−1^K^−1^, which was 94.62 % of the predicted value of the Mexwell-Eucken model. When the diamond particle size was 40 μm, the thermal conductivity of the composite was only 241 Wm^−1^K^−1^. Heat transfer in the diamond/Al composites mainly depends on the phonons formed in diamond, and the interface and pores are main factors affecting thermal conduction [37]. Smaller diamond particle sizes lead to increased interface area, which in turn reduces phonon diffraction and increases phonon heat dissipation. This results in a decrease in thermal conductivity when the amount of diamond is kept constant. While for the 400 μm-diamond/Al composite, the experimental thermal conductivity was only 242 Wm^−1^K^−1^, which was far from the predicted value of the both models. Larger diamond particle sizes result in lower relative density of the composite material, indicating the presence of more voids. This reduces thermal conductivity due to the poor thermal conductivity of air. The porosity of the diamond/Al composites is the main element affecting the thermal conductivity. Since the pores aggravate the phonon scattering and decrease the mean free path of the phonon, the thermal conductivity of the composites is reduced.

#### 3.4.4 Coefficient of thermal expansion

The CTE is also one of the most important performance indicators of thermally conductive materials. The main models used to study the thermal expansion coefficient of metal matrix composites are ROM model, Turner model and Kerner model. The ROM model only considers the proportion of each component in the composite, and its expression is shown in 8:

(8) $$\alpha =\alpha_{Al}V_{Al}+\alpha_{diamond}V_{diamond}$$

which α_Al_, α_diamond_ are the thermal expansion coefficients of the Al matrix and diamond; V_Al_, V_diamond_ are the volume fractions of the Al matrix and diamond.

The Turner model assumes that the stress is static, and the diamond particles are evenly distributed, the expression is shown in 9:

(9) $$\alpha =\frac{\alpha_{Al}V_{Al}K_{Al}+\alpha_{diamond}V_{diamond}K_{diamond}}{V_{Al}K_{Al}+V_{diamond}K_{diamond}}$$

which K_Al_, K_diamond_ are the bulk elastic modulus of Al matrix and diamond.

In addition to the effect of microscopic stress and strain, the Kerner model also considers the effect of material shear force on its internal grain boundaries. The predicted CTE of α is shown in equation (10).

(10) $$\alpha =\alpha_{ROM}+V_{Al}V_{diamond}\left(\alpha_{diamond}-\alpha_{Al}\right)\frac{K_{diamond}-K_{Al}}{V_{Al}K_{Al}+V_{diamond}K_{diamond}+\frac{3K_{diamond}K_{Al}}{4G_{Al}}}$$

which α_ROM_ is the thermal expansion coefficient value calculated by the ROM model, G_Al_ is the shear modulus of the Al matrix.

**Fig. 10:**
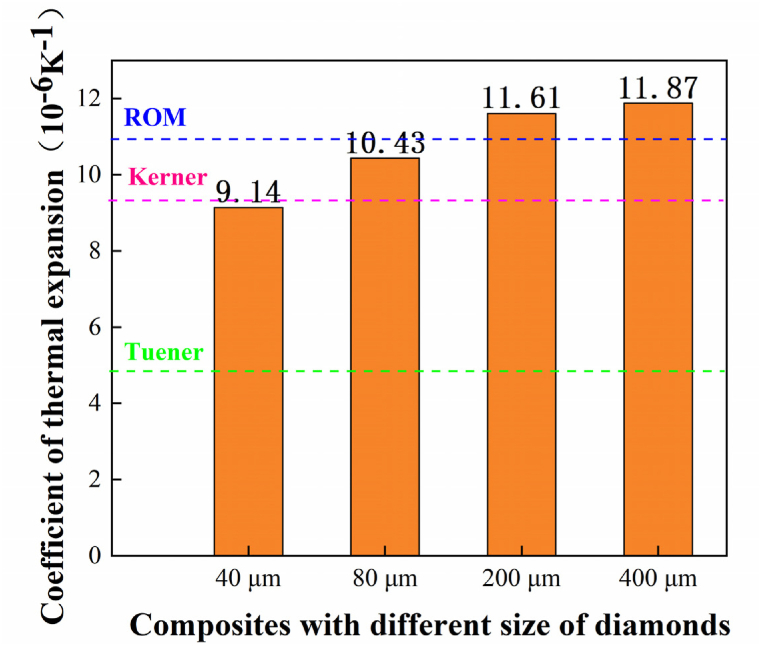
Measured and modelled of thermal expansion coefficient with different particle sizes of diamond.

Fig. 10 shows the comparison between the model predicted values and the actual measured values. It can be seen that when the particle size of the diamond particles was 40 μm, the CTE of the composites was closer to the Kerner model. With the increase of diamond particle size, the thermal expansion coefficient of the composites gradually increased, which was more consistent with the ROM model. When the diamond particle size increased to 200 μm, the CTE of the composites exceeded the predicted value of the ROM model, and the predicted value of the Turner model was significantly smaller. The CTE of diamond/Al composites increases with the increase of diamond particle size. There are various factors affecting the CTE of composites, such as the particle size of diamond and the interface. With the increase of the diamond particle size, the total bonding force between the Al matrix and the diamond decreased, thereby increasing the voids in the composites. These pores played a main factor affecting the CTE of it. So with the increase of diamond particle,the CTE of the composite were increased.

## 4 Conclusions

This research successfully utilized filtration extrusion to produce diamond/Al composites with a high volume fraction of diamond, investigating the influence of filtration extrusion parameters and diamond particle size on composite properties. The key findings are as follows:

(1) Diamond particles exhibited increased densification with extrusion displacement, enhancing the diamond volume fraction within the composites.
(2) The distribution of diamonds during the filtration extrusion process was intricately linked to extrusion force, with a rapid escalation observed as the diamond content reached a critical density. Larger diamond particles necessitated greater displacement for this force increase.
(3) The relative density of the composites increased with the diamond particle size until it reached a peak of 97.49 % for the 80 μm-diamond/Al composite, then started to decline. Thermal conductivity also rose initially and then fell, with a maximum of 299 Wm^−1^K^−1^ for the 80 μm-diamond/Al composite. The coefficient of thermal expansion increased steadily as the diamond particle size increased, with a minimum value of 9.14 × 10^−6^K^−1^ observed for the 40 μm-diamond/Al composites.

## Data availability statement

Data will be made available on request.

## CRediT authorship contribution statement

**Junfeng Zhao:** Writing – review & editing. **Shuohang Yun:** Writing – original draft, Data curation. **Qiulin Li:** Supervision, Funding acquisition. **liang Wang:** Investigation.

## Declaration of competing interest

The authors declare that they have no known competing financial interests or personal relationships that could have appeared to influence the work reported in this paper.
